# Concomitant existence of left atrial ball thrombus and mural thrombus of left appendage in patient with rheumatic mitral stenosis: a case report

**DOI:** 10.11604/pamj.2022.42.197.33771

**Published:** 2022-07-12

**Authors:** Ismail Oughebbi, Said Benlamkaddem, Reda Bzikha, Mustapha Harandou

**Affiliations:** 1 Department of Cardiovascular Surgery, Ghassani Hospital, Fes, Morocco,; 2 Sidi Mohamed Ben Abdellah University, Fes, Morocco

**Keywords:** Mitral stenosis, ball thrombus, left atriam, case report

## Abstract

Valvular heart disease, and in particular, rheumatic mitral stenosis is frequently associated with intra-cardiac thrombus. Moreover, almost all types of thrombus can be founded in the left atrium whereas the ball shaped thrombus remains very rare. The following case report describes a successful surgical management of an unusual case with a concomitant left atrial free-floating ball thrombus and mural one in a patient who has rheumatic mitral stenosis with atrial fibrillation. However, this patient did not present any cerebral or peripheral embolic events. Left atrial ball thrombus is relatively a rare case. Once the diagnosis was made, the surgical removal should be done immediately to avoid embolic complications and deterioration of hemodynamics.

## Introduction

Mitral valve stenosis is at high risks of thromboembolic complications and hemodynamic deterioration. Although, left atrial ball thrombus is very uncommon and it is classified as a specific subgroup of thrombi. Indeed, this kind of thrombus has more catastrophic consequences than the other ones. We report successful surgical management of an unusual case of a concomitant left atrial free-floating ball thrombus and mural one in a 45-year-old patient suffering from rheumatic mitral stenosis with atrial fibrillation.

## Patient and observation

**Patient information:** we report the case of a 45-year-old woman with a history of severe rheumatic mitral stenosis. She was presented to the emergency room for a recent worsening of dyspnea.

**Clinical findings:** within her admission, she was conscious, with elevated jugular venous pulse, normal blood pressure and tachycardia at 115 beats/min, while she didn´t present any neurological symptoms or any signs of stroke. Thus, the electrocardiogram revealed rapid atrial fibrillation.

**Figure 1:**
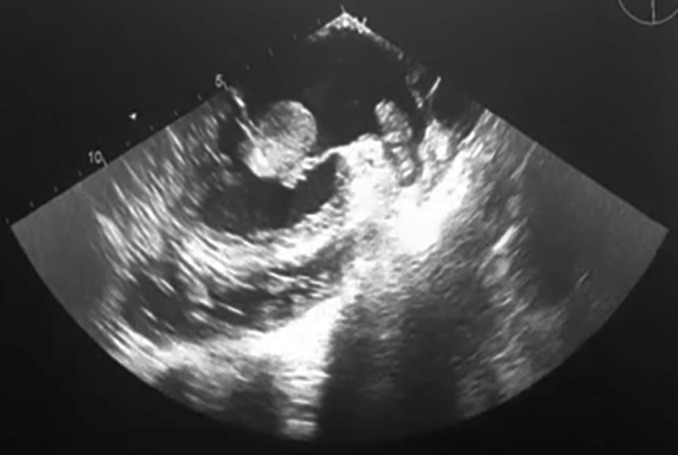
mid-esophageal 4-chambers view showing the free-floating thrombus in the left atrium and the mural one which is adhered to the left appendage

**Diagnostic assessment:** the patient underwent transthoracic echocardiography, which revealed a mobile left atrium mass. In order to identify more precisely the characteristics of the mass, we performed transesophageal echocardiography, which showed that this mass was spherical and smooth shaped, located in the left atrial chamber, free-floating without evidence of any attachment, and measuring 35x25 mm in diameters (Figure 1). This free-floating mass occluded occasionally the stenotic mitral orifice during diastole without passing through. However, the mass was pushed back to the left atrium by the mitral leaflets during systole. Moreover, transesophageal echocardiography revealed a large mural thrombus of the left atrial appendage that was obliviated in transthoracic echocardiography. It also revealed severe mitral valve stenosis with reduced mitral valve area to 0.8 cm^2^, and dilated left atrium.

**Therapeutic intervention:** giving the size, shape and free motion of the thrombus in the left atrium, urgent cardiac surgery was performed within hours. We applied minimal cardiac manipulation throughout the procedure to prevent migration of the ball-thrombus and to prevent “hole-in-one thrombus” effect. After sternotomy, cardiopulmonary bypass (CPB) was started in standard fashion by aortic and bi-caval cannulation. Cardiac arrest and myocardial protection were obtained by the use of antegrade intermittent crystalloid cardioplegia and topical ice-cooling. Then, the left atrial vent was placed after cross-clamp to avoid a thrombotic event. The thrombi and valve replacement were achieved via the transseptal approach, as we always manage to check the tricuspid valve when on left cardiac surgery.

**Figure 2:**
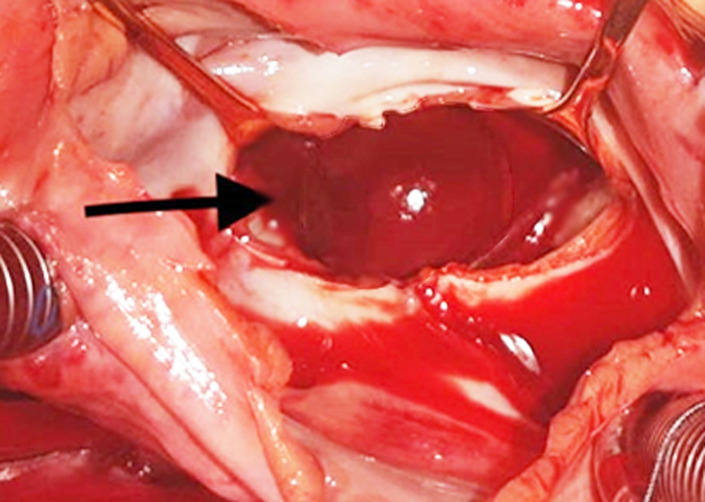
operative view showing the mass in the left atrium; the surgical specimen is a spherical mass with a smooth surface

**Figure 3:**
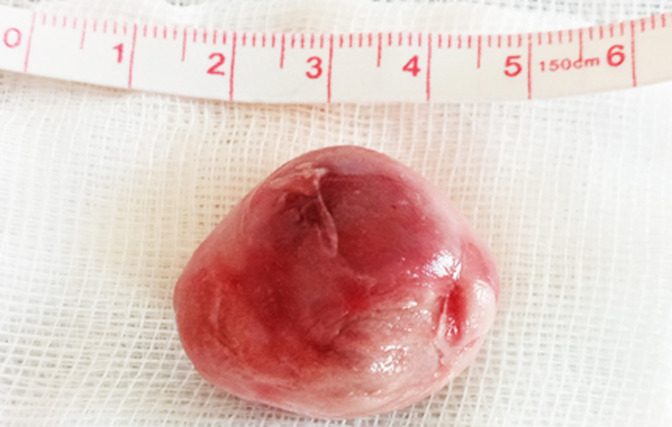
the free-floating thrombus ball removed from the left atrium

After cardiotomy, the left atrium was found to be enlarged and (presence of) a round, smooth, nonpedunculated thrombus measuring 35 mm in diameter was found within floating freely in the cavity of the atrium (Figure 2). In addition, we found another thrombus adherent to the left appendage. The intervention consisted of the removal of the spherical mass from the left atrium, thrombectomy of left atrial appendage with its endocardial exclusion, and mitral valve replacement with a mechanical prosthesis. Indeed, no attachment of the mass was found at operation (Figure 3). Histopathological examination of the ball thrombus showed characteristic ‘lines of Zahn´ confirming it as thrombus.

**Follow-up and outcomes:** the postoperative course was uneventful, anticoagulation was resumed the second postoperative day, and the patient was discharged on the tenth day.

**Patient perspective:** during the time she was hospitalized and after the surgery, the patient was delighted with the care she received and was optimistic about the outcome of her condition.

**Informed consent:** written informed consent was obtained from the patient for publication of this case report and accompanying images.

## Discussion

“Ball thrombus” is defined by Wood [1], as an unattached clot that its cross-sectional diameter is greater than the orifice of the chamber where it is present. It was identified for the first time in 1814 by the autopsy findings of a 15-year-old girl who had rheumatic mitral stenosis. Since then, several cases have been reported.

In the present case, the risk factors of the creation of clots are the presence of severe rheumatic mitral stenosis and atrial fibrillation. Nevertheless, other potential risk factors that had been reported in several case reports are status-post mitral valve replacement with mechanical or biological prosthesis, mitral valve repair, aortic valve replacement, hypertrophic cardiomyopathy, and chronic nonvalvular atrial fibrillation [2]. As reported by Raddino *et al*. the existence of the mural thrombus is crucial for the formation of the free-floating thrombus [3]. In our case, we believe that the thrombus-ball was initially attached by a pedicle to the other one which is adhered to the left atrial appendage. Then, as the ball-thrombus became larger, the pedicle seemed thinner and let the ball thrombus break free. The mechanism of the typical spherical shape, is that the layers of fresh clot was formed progressively as it is spinning within the atrium [4]. Histologically, the thrombus was covered by endothelium, thus suggesting that this kind of thrombus is more resistant to anticoagulation and dissolution.

In order to diagnose the free-floating thrombus of the left atrium using the echocardiography, one of two criteria must be present, the first one is that the left atrium thrombus must be bigger than the cross-sectional area of the valve orifice, and the second one is that it should have a smooth surface without any attachment to the atrial wall or the mitral valve. Both the criteria were present in our case report. So, transesophageal echocardiography remains the exam of choice to diagnose this mass, to follow-up the patients under pharmaceutical treatment, and to manage the surgery. The left atrial ball-thrombus may lead to severe complications [5]. The fatal one is sudden death by the obstruction of the mitral orifice, known as the “hole-in-one thrombus” effect [6]. The other one is systemic embolization, which results from defragmentation of the ball-thrombus or of its pedicle during the formation phase.

Once the diagnosis of a free-floating ball thrombus is established, emergency surgery is recommended in order to prevent complications. Thus, the surgical removal of the thrombus with concomitant treatment of the cause is the gold standard of therapeutical management [7]. Therefore, once the detection of a ball thrombus in the left cavity is made, urgent surgical removal is indicated, especially when one of the following echographic criteria are present: surgical mitral valve disease; enlarged or giant left atrium; atrial fibrillation; complete mobility; diameter greater than 1cm; multiple ball-thrombi; and left ventricular location (“hole-in-two thrombi” effect) [6-8].

## Conclusion

Left atrial ball thrombus is rare. Once the diagnosis is made, the surgical removal should be done immediately to avoid embolic complications and deterioration of hemodynamics.
